# Ear-Bot: Locust Ear-on-a-Chip Bio-Hybrid Platform

**DOI:** 10.3390/s21010228

**Published:** 2021-01-01

**Authors:** Idan Fishel, Yoni Amit, Neta Shvil, Anton Sheinin, Amir Ayali, Yossi Yovel, Ben M. Maoz

**Affiliations:** 1 School of Zoology, Tel Aviv University, Tel Aviv 6997801, Israel; idanfishel@mail.tau.ac.il (I.F.); yoniamit@tauex.tau.ac.il (Y.A.); shviln@mail.tau.ac.il (N.S.); santon@post.tau.ac.il (A.S.); ayali@tauex.tau.ac.il (A.A.); yossiy@tauex.tau.ac.il (Y.Y.); 2 Department of Biomedical Engineering, Tel Aviv University, Tel Aviv P.O. Box 39040, Israel; 3 Sagol School of Neuroscience, Tel Aviv University, Tel Aviv 6997801, Israel; 4 School of Mechanical Engineering, Tel Aviv University, Tel Aviv 6997801, Israel; 5 The Center for Nanoscience and Nanotechnology, Tel Aviv University, Tel Aviv 6997801, Israel

**Keywords:** bio-robot, bio-hybrid, acoustic response, locust robot, ear-on-a-chip

## Abstract

During hundreds of millions of years of evolution, insects have evolved some of the most efficient and robust sensing organs, often far more sensitive than their man-made equivalents. In this study, we demonstrate a hybrid bio-technological approach, integrating a locust tympanic ear with a robotic platform. Using an Ear-on-a-Chip method, we manage to create a long-lasting miniature sensory device that operates as part of a bio-hybrid robot. The neural signals recorded from the ear in response to sound pulses, are processed and used to control the robot’s motion. This work is a proof of concept, demonstrating the use of biological ears for robotic sensing and control.

## 1. Introduction

Over the course of hundreds of millions of years of evolution, insects have developed ingeniously simple but sensitive sensors, which are small, lightweight, adaptable to extremely varied environments, characterized by low power consumption, and surpass many manmade, artificial sensors. These unique properties have made these biological sensors very appealing for utilization in technological applications. There are, however, a number of significant challenges in our way to exploiting them, including, (1) the need to dissect the sensor from the insect, keeping its integrity and functionality; (2) the need to extract and process the sensor-generated biological signal; (3) the need to integrate the biological sensor into the technological platform (e.g., a mobile robot); and (4) the need to prolong the life of the biological sensor in order to allow extended use of the technology. 

These challenges have been met in recent years by the novel field of bio-hybrid organisms, demonstrating the ability of integrating the outstanding capabilities of biological sensors with the capabilities of electronic hardware and autonomous technological innovations, such as robots [1]. Since the presentation of the first bio-hybrid robot, which used the silkworm moth’s antenna to measure pheromone concentration in the air [2], other bio-hybrid robots were developed, utilizing various biological sensors in order to provide input to robotic platforms. These include the use of olfactory systems to identify the source of odorants [3,4,5,6], and even to distinguish between odorant concentrations emitted by explosives [7]; photoreceptors, which are used for optical guidance [8,9]; and even a bio-inspired light-guided swimming robot that mimics a ray fish and was powered by rat heart muscle cells [10]. To the best of our knowledge, none of these examples to date, used an insect auditory system.

Hearing evolved multiple times in insects [11] serving the functions of scene analysis and communication. Insect auditory sensors are highly diverse (1) in morphology—insect ears range from near-field sensitive antennae to far-field sensitive tympanic membranes^11^; (2) in function—ranging from narrow band filters (e.g., in mosquitos [12]) to wide-band sensors (e.g., in noctuid moths [13]) and (3) in neural processing—ranging from one neuron ears (e.g., in noctuid moths) to ears with thousands of intervening neurons (e.g., 15,000 in the male mosquitos). Several recent studies applied bio-inspired phonotaxis in robots, equipped with acoustic electronic sensors used to identify and respond to sound [14,15,16,17,18,19]. However, until now, no study demonstrated a bio-hybrid robotic platform, which is able to respond to sound via a biological sensor (e.g., an ear). Utilizing the biological sensor (the ear) on a robotic platform is of course challenging. However, it allowed us the advantage of being able to compare its behavior and capacity to that of the well-characterized natural locust ear, on the one hand, and to a purely technological device (microphone), on the other. 

**Figure 1:**
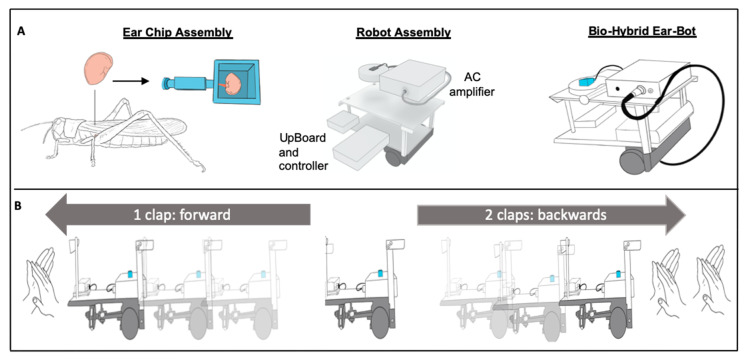
The establishment of the Ear-Bot (**A**) Left to right: The tympanal membrane of the locust ear and its position on the animal’s body; locust auditory organ inside a special chip designed for best electrophysiological recording and later to be attached to the mobile platform; Ear-chip attached to the mobile platform creating the Ear-Bot. (**B**) The Ear-Bot response to sound. Once the Ear-Bot identifies one sound (clap) it goes forward, and once the Ear-Bot identifies two sequential sounds (claps), it moves backwards.

In this work, we developed an Ear-Bot, a bio-hybrid platform which integrates the hearing system of the desert locust as a sensing input, interfaced with a moving robotic platform. We chose the locust’s tympanal organ (ear), as it is sensitive to a wide range of frequencies [20], very well characterized [21], and serves as a good model for electrophysiological readouts from the nervous system. Moreover, in order to tackle the above-mentioned challenges, we used recent developments in micro-physiological systems (MPS), also known as Organs-on-a-Chip (OoC) [22,23] in order to create a modular tissue support, and a custom algorithm for signal analysis. To this end, we isolated and characterized the ex vitro locust hearing system (Figure 1a) and integrated it with a custom 3D microfluidic chip. The chip was designed in such a way to enable the long-term viability of the ear while allowing it to be placed on top of a moving small robotic platform (Figure 1a, Figures S1 and S2). It was further equipped with custom-made electrodes, which enabled measuring the electrophysiological response of the ear and transmitting it to the robot. The robot included all the electronics (pre-amplifiers, board, etc.) necessary to process the signal and run different algorithms in order to control the robot accordingly (Figure 1b). Overall, in this work, we present an integrated bio-hybrid platform, which uses the locust Ear-on-a-Chip, as an auditory sensor for controlling a robot. 

## 2. Materials and Methods

### 2.1. Animals

All experiments were performed on adult desert locusts, *Schistocerca gregaria*, of both sexes, from our breeding colony at the School of Zoology, Tel Aviv University, Israel. The locusts were reared over many generations in 60-liter metal cages at a density of 100–160 animals per cage, under a controlled temperature of 30 ^◦^C, 35–60% humidity, and a 12 D:12 L cycle. Additional radiant heat was provided by 25 W incandescent electric bulbs during daytime to reach a final day temperature of 35–37 ^◦^C. The locusts were fed daily with wheat seedlings and dry oats.

### 2.2. Preparation

An ex vitro preparation including the tympanal organ and the auditory nerve (nerve VI) was prepared as follows: Locusts were anesthetized with CO_2_, decapitated, and legs and wings were cut. After excising the abdomen posteriorly to the fourth abdominal segment, the thorax was opened along the dorsal midline, pinned to a clean Sylgard dish (Sylgard 182 silicon Elastomer, Dow Corning Corp., Midland, MI, USA), dorsal side up, and bathed in locust saline (in mM: 150 NaCl, 5 KCl, 5 CaCl_2_, 2 MgCI_2_, 10 Hepes, 25 sucrose at pH 7.4). Gut, fat tissue, air sacs, and cuticular parts were carefully removed with fine forceps, exposing the third (metathoracic) thoracic ganglion. Next, a window of cuticle including the tympanal organ was carefully cut free. Finally, the auditory nerve was identified, cleaned, cut close to its origin from the thoracic ganglion, and, together with the tympanal organ, dissected out of the body cavity and put in a special designed chip filled with locust saline. 

### 2.3. Chip Design and 3D Printing

The “ear” chip was designed to support long-term viability and functionality of the isolated sensory tissue (Figure S2). Several designs were fabricated and tested during the study. The different chips were designed via SolidWorks CAD software (SolidWorks Corporation, MA, USA), then fabricated using 3D printed (Form2 SLA 3D printer, Formlabs) biocompatible dental long-term (LT) clear resin (Formlabs) (Figure S1). Prior to testing, the fabricated chips were cleaned and UV sterilized (Form wash & Form cure, Formlab). 

### 2.4. Electrophysiological Recordings and Sound Stimulation

We recorded extracellularly the activity of the auditory nerve using custom-made suction electrode. Borosilicate glass capillaries (1.5 MM X 86 MM,4″, A-M systems) were pulled with a P87 puller (Sutter Instruments, Instruments, Novato, CA, USA). Signals were amplified 1000 times by four-channel differential AC amplifiers (Model 1700, A-M Systems, Bellevue, WA, USA), then filtered with a 300 Hz high-pass filter and a 5 kHz low-pass filter. This amplified and filtered data were sampled at 20 kHz by Digidata (1440 A 16bit, Axon CNS) and AxoScope 10 software (Axon™ pCLAMP™) running on Windows v10. 

The activity of the auditory nerve when mounted on the mobile recording system (Robot) was extracellularly recorded by the same custom-made suction electrode and amplified using a custom-designed AC amplifier (see below) (Movie S1).

Sound stimulation of the preparation was programmed using Avisoft software (sas-lab lite) and delivered by a speaker (Cyber Acoustics) which was placed on a special custom-made stand (Tel Aviv University workshop). This stand enabled flexibility in the speaker’s angle (0°–180°) and distance (5 cm–30 cm) in respect to the tympanal Ear-on-a-Chip (Figure S3, Movie S2). A reference microphone was positioned in proximity to the chip and the speaker to be used as positive control.

The stimulation protocol was composed of 60 dB (decibels) sound pressure level (SPL) (Relative 10 cm), three 500 ms long sound pulses, spaced by 1000 ms of silence as commonly done for auditory brainstem response (ABR) [24]. Each group of three pulses was of different frequency (between 0.2 kHz and 15 kHz, see Figure S4) and separated from the previous group by 5000 ms. Sound intensity was normalized to be equal in all frequencies by compensating for the speaker’s frequency response.

### 2.5. Custom-Designed AC Amplifier for Neural Recordings

The amplifier has the following characteristics (Figure S6): bandwidth 150 Hz–1052 Hz, gain 1044 or 2089 (selectable), common-mode rejection ratio (CMRR) —minimum 96 dB. The initial cascade or headstage (U1) was based on INA121 instrumentation amplifier (Texas Instruments). In order to keep the high value of the CMRR, the headstage was DC coupled to the suction electrode. This allowed us to utilize the manufacturer’s specified value of the INA121 CMRR (minimum 96 dB). The gain of the headstage was set to 10 with the gain-setting resistor R1. The DC offsets usually arising at the metal electrodes was removed by the passive high-pass filter at the output of the headstage (C3, R2; cutoff = 150 Hz) and amplified by the second stage (U2.1, LM358, Texas Instruments). The gain of the 2nd stage was set to 3.9. In its turn, the output signal of the 2nd stage low-pass filtered and additionally amplified by the 3d stage (U2.2, LM358). The cutoff frequency of the low-pass filter of the 3d stage was 1052 Hz; the gain was manually selectable (either 27 or 54). As a result, the total gain of the amplifier was either 1044 or 2089, depending on the position of the “Gain” toggle switch. The power for the amplifier was provided by the 9 V battery; the bipolar supply was created with the help of simple resistive divider (R7, R8). All resistor tolerances were at least 1%, all capacitor tolerances were at least 5%. The amplifier was mounted in a shielded aluminum enclosure (Hammond Manufacturing).

### 2.6. The Robot

The robot comprised of an Intel UpBoard, an adc converter (USB-6211, National Instruments), a micro Controller "D1 Mini" (ESP8266 D1 Mini WiFi Dev Board, Addicore) used to control 2 DC motors that controlled the wheels. The Intel Upboard ran a standard windows 10, and ran an algorithm written in python (3.0, Python Software Foundation©). The algorithm thresholded the signal and counted how many peaks are in the thresholded signal in a sliding time window of 2 s. The robot was programed to move forward when detecting one sound pulse and backwards when detecting two.

### 2.7. Data Analysis

Overall, 29 experiments were performed in this study on each of the locust tympanal organs, each employing between 12 and 36 stimulus repetitions, to finally obtain ~1836 recordings of the auditory nerve spike response pattern.

The data from the nerve recordings were processed using DataView software (V.11, University of St.Andrews). For every single stimulus, the response magnitude was calculated as follows: a 0.2 × 10^−5^ threshold was set to detect the sound stimulus and set it symmetrically to 500 ms. Next. A filter was used (Finite impulse response (FIR), bandwidth 1 Hz–1000 Hz) on the nerve electrical response, and the number of spikes were counted within the stimulation window. This allowed us to calculate the spike frequency of the electrophysiological response. The analysis was based on the identification of spike (action potential) events only, and not bursts. The spikes were detected and identified by their amplitude using mean standard deviation (SD) threshold source (mean +/− SD × 7).

Cross correlation: The correlation between the speaker stimuli and the tympanal organ responses was tested in MATLAB (The Mathworks, Inc.R2019b), utilizing the Cross correlation function with maximum lag of 1000 ms. Data was plotted in GraphPad Prism 5 (GraphPad software Inc., San Diego, CA, USA).

Statistics: P-values were calculated using ANOVA followed by Tukey’s post hoc test (GraphPad Prism 5, GraphPad software Inc., San Diego, CA, USA).

## 3. Results

The establishment of the Ear-Bot required multiple steps, which needed to be integrated onto one biocompatible, mobile platform, to enable the dynamic response to sounds (Figure 1). In the following sections we will elaborate on the development and establishment of the Ear-Bot.

### 3.1. Establishing the Ear-on-a-Chip

**Figure 2:**
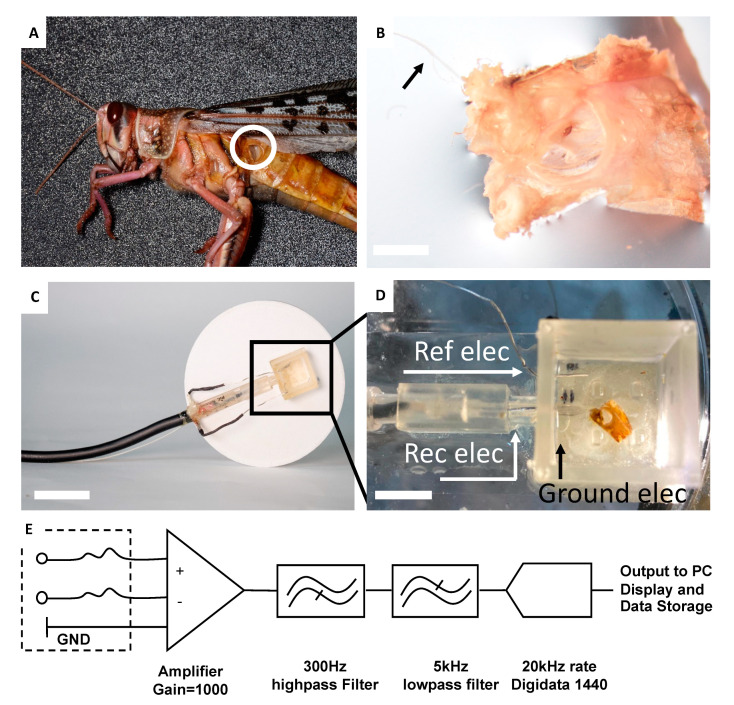
Characterization of the Ear-Chip response to sounds (**A**) The locust’s tympanal organ (**B**). Isolated locust’s tympanal organ with an intact auditory nerve (black arrow). Scale bar 0.1 cm (**C**) A microfluidic chip connected to a custom designed suction electrode. Scale bar 2 cm (**D**) Locust auditory organ inside a special chip designed for best electrophysiological recording. The locust auditory nerve sucked into the suction electrode prior to recording. Scale bar 0.6 cm. Ref elec: *Reference electrode*, Rec elec: *Recording electrode*, Ground elec: *Ground electrode*. (**E**) Diagram of the electrical connections of the Ear-Chip.

The Ear-on-a-Chip is based on the integration of the locust ear (Figure 1A, Figure 2A,B) with a microfluidic chip (Figure 1A, Figure 2C,D, Figure S3). The locust ear was isolated in such a way as to maintain the integrity of both the air sac and the intact auditory nerve (Method section, Figure 2A,B), in order to ensure the full range of sound detection, while preforming the electrophysiological measurements. The microfluidic chip (Figure 2C,D, Figure S1) was designed to answer the need for an aquatic environment suitable for sustaining the nerve and ear, together with the necessity to allow access to air and sound pressure. The Ear-Chip was also designed to meet the challenge of consistent and stable electrophysiological measurements, together with the need for mobility. Unlike standard electrophysiological recordings that call for a solid stationary platform, the robot is a light, mobile platform. After testing several options of integrated extracellular recording apparatuses, the Ear-Chip was equipped with a custom-made suction electrode. The electrode provided good answers to the above requirements, including consistent and stable connection with the nerve, allowing the long term recording of the electrophysiological activity of the ear, in the chip (Figure 2D, Figure S2, Movie S1). 

**Figure 3:**
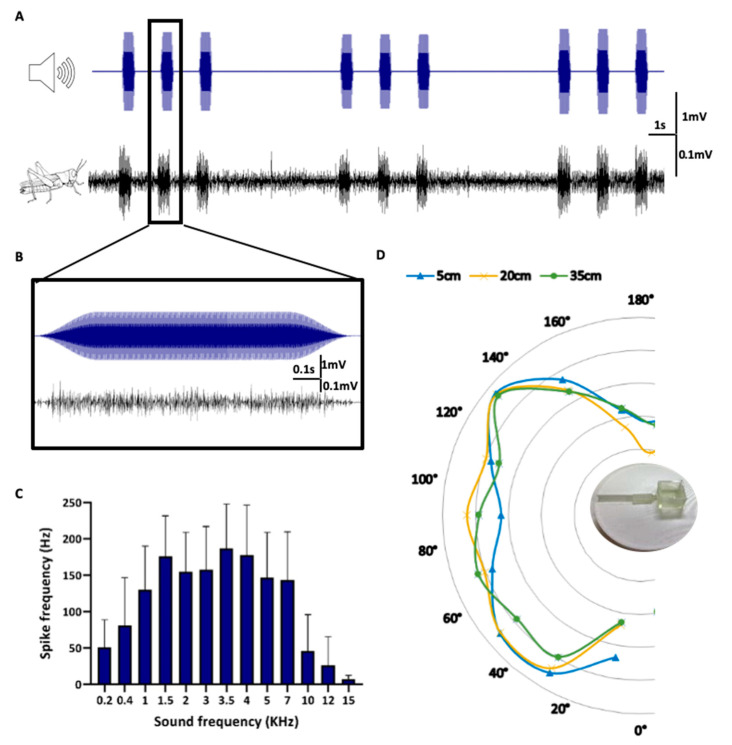
Characterization of the Ear-Chip response to sounds (**A**). Locust hearing organ’s electrophysiological responses (Black, Bottom) to sounds produced from a speaker (Blue, Top) (**B**). Zoom in to one of the pulses. (**C**) Spike frequency of the sensor response to different sound frequencies. 3.5 kHz seems to trigger the strongest response (n = 6), cross correlation analysis presented in Figure S5. (**D**) Spike frequency of the sensor response to sound in 3.5 kHz from different angles and distances. The sensor shows response to a range of angles for all distances tested with no significant difference (n = 6). Spike frequency of the sensor response represented by the distance of the line from the center of the circle.

In order to validate and optimize the Ear-Chip, the ear was stimulated as described in the method section. (Method section, Figure 3A,B, Figures S4 and S5). The sound-induced electrical activity was measured and correlated with the applied stimulation. As shown in Figure 3A,B, and Movie S1, the Ear-Chip enabled consistent transformation of the sound signals to electrical activity, well reflecting the in vivo case. As a next step, we characterized the response of the Ear-Chip to different sound frequencies, distances, and directions of the sound source. This was done in order to further establish both the integrity and functionality of the removed-ear and to validate the design of the Ear-Chip (to ensure that it does not affect the ear’s response to sound). As shown in Figure 3C, the locust Ear-Chip demonstrate similar frequency-dependent responses, as those reported in the literature for the locust hearing system [20]. It can be seen that the best response is around 3.5 kHz (±2) and that the response decays beyond 7 kHz, which can also be seen in the cross correlation analysis in Figure S5. To ensure that the chip does not interfere with the spatial response of the ear, we characterized the response of the Ear-Chip to sounds at different frequencies from different angles (Figure 3C,D). This was conducted via a custom made system which enabled moving the sound source radially around the Ear-Chip, while also modifying the distance from the Chip as shown in Figure S3. As excepted, there was no significant change in the Ear-Chip response to sound from the different directions, and no difference was observed in the response to sound from distances between 5 and 35 cm. These characterizations ensured that our Ear-bot will be able to react to sound from different directions, and distances. 

### 3.2. Establishing the Robot

**Figure 4:**
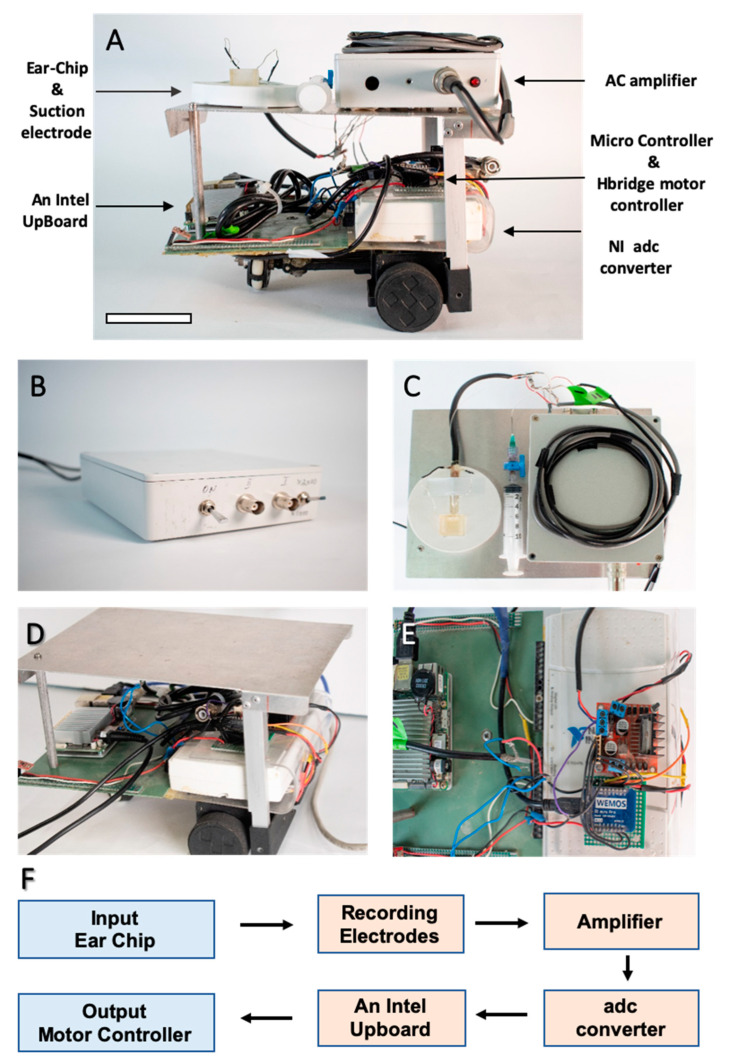
The robot and its components (**A**) Robot overview from the side. Scale bar 6 cm (**B**) Custom-designed AC amplifier (**C**) Robot top view shows the electrophysiological measuring system (EMS). The EMS includes the customized chip, the suction electrode, and the signal amplifier (**D**) The controller and signal processor system (CSPS). (**E**) The CSPS is composed from two parts, the signal processor (bottom), and the controller (on top of it). (**F**) Logic flow chart of the electrical components. A detailed diagram is found in Figures S6 and S7.

The robot (Figure 4A) can be divided into two main components: (1) the electrophysiological measuring system (EMS) (Figure 4B,C), and (2) the controller and signal processing system (CSPS) (Figure 4D,E). The EMS was composed of the electrode (within the Ear-Chip), which was connected to an amplifier with selectable gain (1044 or 2089) (Method section, Figure 4B,C, Figure S6). The system allows to amplify the electrophysiological signals so that the CSPS will be able to process them. The CSPS is composed of two parts, the signal processor, and the controller (Figure 4D,E). Signal processing included a peak detection algorithm to identify the number of distinct sound events (e.g., claps). The controller then controlled the motors accordingly. The robot’s response to sound was first tested by generating different sounds directly, via a connected microphone, and identifying the different motor responses of the robot. In our test, one hand clap caused the robot to move forward (to the left of the image) and two claps caused the robot to move backwards (to the right of the image) (data not shown). 

### 3.3. Bio-Hybrid Ear-Bot Integration and Assessment 

**Figure 5:**
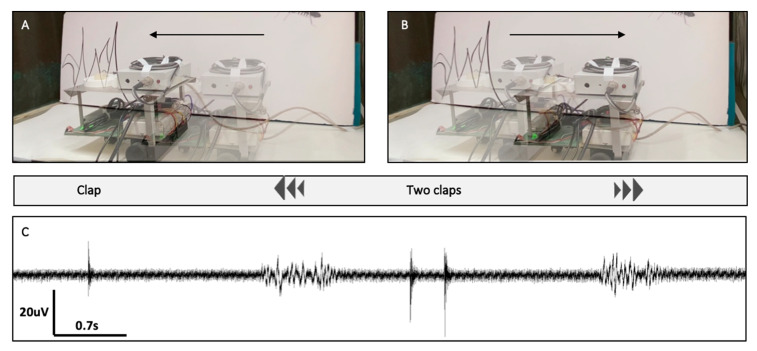
Hybrid Bio-Robotic-Sensing system. (**A**) The Ear-Bot moves forward as a response to one clap and (**B**) backward as a response to two claps. (**C**) The Ear-Chip electrophysiological response to the claps.

Once the robot was developed and validated using a regular microphone, the input sensor was replaced with the biological hearing system (i.e., the Ear-Chip), to create the Ear-Bot. As shown in Figure 5 (Movie S3), the Ear-Bot’s response to sound was similar to that demonstrated with the microphone as input. Once a clap was made, the locust ear identified the sound and converted it to an electrical signal that was transmitted to the EMS and CSPS which controlled the robot according to the number of claps. Importantly, the system was capable of distinguishing between the inherent noise of the robot due to the motors, (see around 5 s in Figure 5) and the human made noise (clap). As was shown in Figure 3C, the ear was sensitive to a wide range of frequencies, and therefore can respond to a verity of sounds. 

## 4. Discussion

The presented work constitutes a proof-of-concept study, demonstrating a novel bio-hybrid system based on a locust Ear-on-a-Chip and a mobile robotic device for the detection and identification of auditory signals. We have described the development of the Ear-Bot as a process that required the integration of different components. We note the importance of the following points, to ensure the proper functionality and success of the Ear-Bot. 

The locust hearing system: as Figure 3c shows, the ear can respond to a wide range of frequencies. It is important to keep in mind, however, that the ear is a biological system, i.e., its functionality may be both age- and sex-dependent. In this study, in order to facilitate the ear extraction, we used young adult locusts (1–2 weeks post-maturity), as at this age, the (full size) locust is relatively low in fat reserves, allowing easier access to the nervous system and simpler removal of the hearing system. A second advantage of the young-adults is their reported better response to low frequencies, together with shorter latency at the neural response [25]. Last, regarding sex differences, it was found that after four weeks post-adult females are less sensitive to sounds, but there are no significant differences before that [25]. 

Another significant factor that should be taken into account is caution during the ear isolation process. It is extremely important not to damage the air sac (an extension of the trachea) as it might affect the ear’s response to low frequencies [26]. Other points that should be noted, are the environment the ear is experiencing and the time frame of assembling the system, as both might significantly affect the ability to measure the electrical activity of the ear. The auditory nerve is very sensitive and needs to be kept in an aquatic environment at all times. Therefore, once the ear was isolated, it was immediately placed in a saline buffer. 

While, as noted, the locust hearing system offers major advantages. Future work can also test and apply ears from insects other than the locust, which may allow different acoustic capabilities.

The chip: Contrary to regular electrophysiological measurements, which take place in a static, stable platform, a robot is a mobile platform, which requires a custom holder for the ear, allowing the consistent recording of the electrophysiological activity from the ear, while the robot is moving. In relation to these multiple challenges, we found that interfacing the nerve within the chip with fixed wire electrodes did not allow the stability needed for consistent measurement of the electrophysiological signal from the ear. It turned out that this was better provided by the flexibility allowed by a suction electrode. An important parameter is the need to keep both the viability and functionality of the ear in vitro for at least a couple of hours (Figure S2). To face this challenge, we designed the chip in such a way as to include four dedicated pillars (Figure S1) which keep the ear in place, allow it to be exposed to air (to receive sound), while also allowing medium circulation for ensuring the ear viability. 

During the Ear-on-a-Chip characterization, we show a clear difference in the ear’s response to different frequencies (Figure 3c). However, we did not observe a significant change in the Ear-Chip response to different angles and distances (Figure 3d). While one can expect such differences, two factors should be taken into account: (1) The range of distances that were tested in this work was limited, as we wanted to test the response to sound within the setup that the Ear-Bot will operate in. (2) The geometry and design of the chip was such that did not provide major discrepancies between the direction the sound comes from. Further work is required to establish this point. It might simply require a set of two ears (it is still unclear whether the locust can identify the direction of sound with only one ear, as in most cases, two ears are needed [27]). 

The robot: The mobile robotic platform was designed and built in such a way as to be suitable for carrying a number of Ear-on-a-Chip systems, and to enable the integration of multiple signals in order to have directional hearing (for future studies). This feature is extremely important if one is interested in navigation by sound. This capability is not supported by the design and size of the platform only, but it is also based on the components being designed in such a way as to allow more than one Ear-Chip to be integrated to the EMS. Nevertheless, the robot was built from “on the shelve components”, which are very accessible and not expensive. This makes the Ear-Bot very reachable to other researchers who are interested in establishing such Bio-Hybrid platform in their laboratory. 

Another point that should be considered, is the robot’s response to sound. In order to facilitate the response to sound, one should try to minimize the noise that is created by the environment, including the noise that is created by the robot itself. Moreover, while we developed and used an algorithm which identifies the number of acoustic events, and responds accordingly; there are other responses that can be chosen, which are based on the sound amplitude, frequency, etc. 

Future applications: This work present a proof of principle of a Bio-Hybrid platform, which integrates an ex vivo system with a robotic platform. This concept can be further developed in order to use other biological sensing systems, which are much more advanced than humanmade sensors (e.g., olfactory system). Furthermore, this concept can be used to combine a number of (similar as well as different) biological sensors to create an advanced bio-robot, which will eventually be able to process various biological inputs and process them, similarly to a living organism. Such capabilities will open novel prospects for the utilization of such platforms for advance sensing of explosives, chemicals, and even earthquakes, matching or even exceeding the capacity of some animals.

## 5. Conclusions

In this work we demonstrated the integration of a biological hearing system as an auditory input to a robotic system, creating a bio-hybrid Ear-Bot. To ensure modularity, stability, and the ability to measure the electrophysiological response of the locust ear, a special microfluidic chip was developed, and presented as Ear-Chip. The integrated platform demonstrated similar auditory responses to sound frequencies as those attributed to the locust ear in vivo, and it enabled the identification and discrimination of the recorded electrophysiological activity by the robotic platform. Furthermore, the integrated Ear-Bot platform demonstrates that a biological hearing system can serve as an auditory sensory input to direct a robot. This platform presents a new ability of bio-hybrid robots that opens the way for the use of a variety of biological auditory systems, which are extremely diverse in their capabilities, as input for bio-hybrid robotic systems.

## Data Availability

The data presented in this study are available on request from the corresponding author.

## Supplementary Materials

The following are available online at https://www.mdpi.com/1424-8220/21/1/228/s1, Figure S1: The Ear-Chip, Figure S2: Stability analysis, Figure S3: Custom platform for assessing the Ear-Chip spatial response to sound, Figure S4: The sound stimulation, Figure S5: Schematics of the AC amplifier, Movie S1: Ear-Chip response to sound, Movie S2: Custom platform for assessing the Ear-Chip spatial response to sound, Movie S3: Ear-Bot response to sound.
