# Supplementary material for: Ear-Bot: Locust Ear-on-a-Chip Bio-Hybrid Platform
Source: Sensors (Basel). 2021 Jan 1;21(1):228. doi: 10.3390/s21010228 (PMC7795996; doi:10.3390/s21010228)
Supplement: Supplementary file 1 [file sensors-21-00228-s001.zip › Sup files/Earbot_Fishel_SI_FINAL.pdf]

sensors

Supporting information for:

Ear-Bot: Locust Ear-on-a-Chip Bio-hybrid Platform

Idan Fishel[1, 2], Yoni Amit[1*], Neta Shvil[1, 3*] Anton Sheinin[1], Amir Ayali[1, 3,#], Yossi Yovel[1, 3,#] and Ben M. Maoz [2,3,4#]

[1] School of Zoology, Tel Aviv University, 6997801 Tel Aviv, Israel; idanfishel@mail.tau.ac.il (I.F.); yoniamit@tauex.tau.ac.il (Y.M.); shviln@mail.tau.ac.il (N.S.); ayali@tauex.tau.ac.il (A.A.); yossiy@tauex.tau.ac.il (Y.Y.)
[2] Department of Biomedical Engineering, Tel Aviv University, PO Box 39040 Tel Aviv, Israel;
[3] Sagol School of Neuroscience, Tel Aviv University, 6997801 Tel Aviv, Israel (A.S.) santon@post.tau.ac.il
[4] School of Mechanical Engineering, Tel Aviv University, 6997801 Tel Aviv, Israel
[5] The Center for Nanoscience and Nanotechnology, Tel Aviv University, 6997801 Tel Aviv, Israel

† Authors have equally contributed.
* Correspondence: bmaoz@tauex.tau.ac.il

Received: date; Accepted: date; Published: date

Figure S1

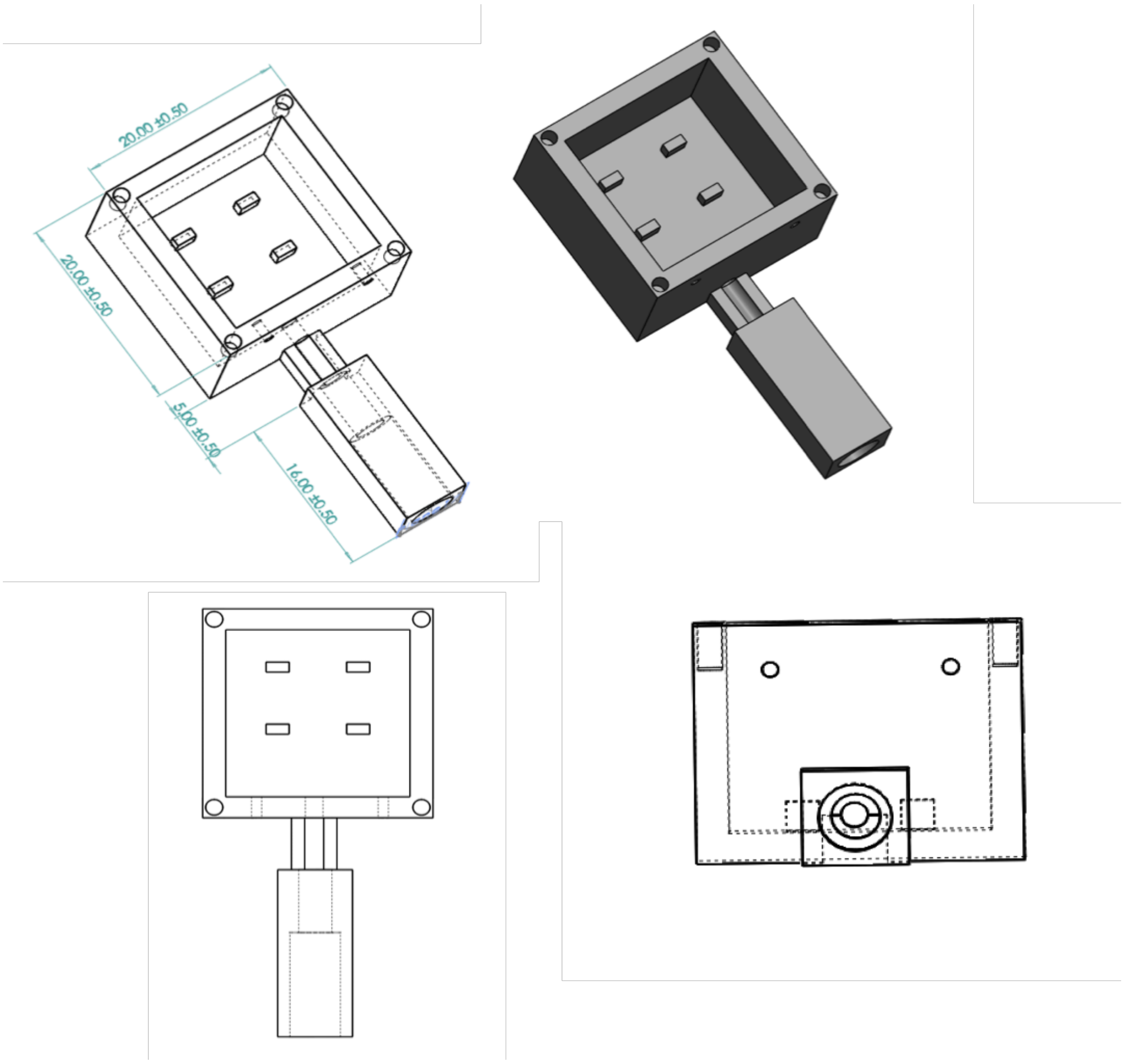

Figure S1: The Ear-Chip

CAD schematics of the Ear-Chip.

Figure S2

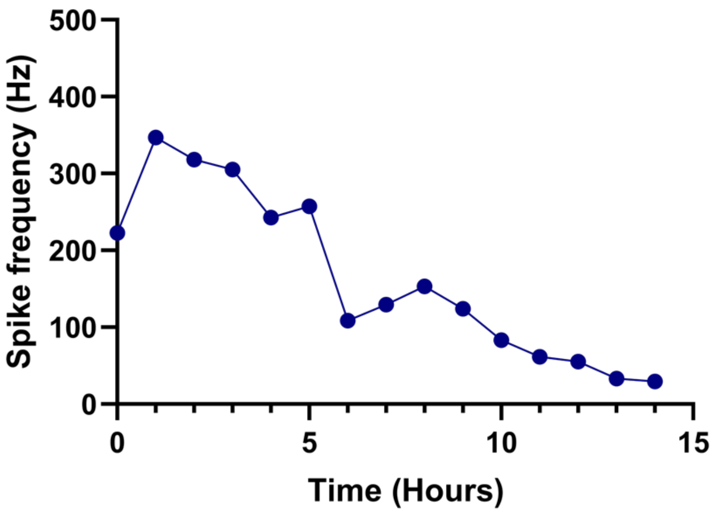

Figure S2: Stability analysis

Stability analysis of a single ear responding to 1kHz sounds for several hours.

Figure S3

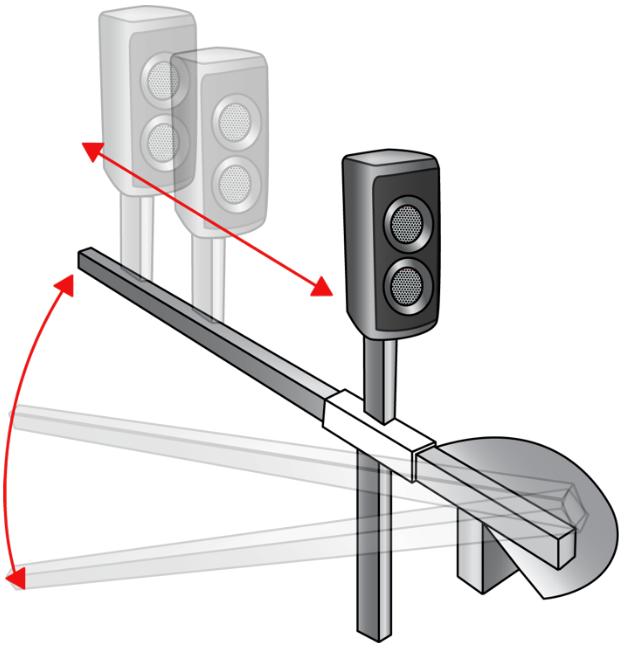

Figure S3: Custom platform for assessing the Ear-Chip spatial response to sound.

A custom-made platform used to characterize the Ear-Chip response to sounds in different frequencies and different angles. The system custom holder enables to move the speakers both radially around the Ear-Chip and modify the distance from the Chip.

Figure S4

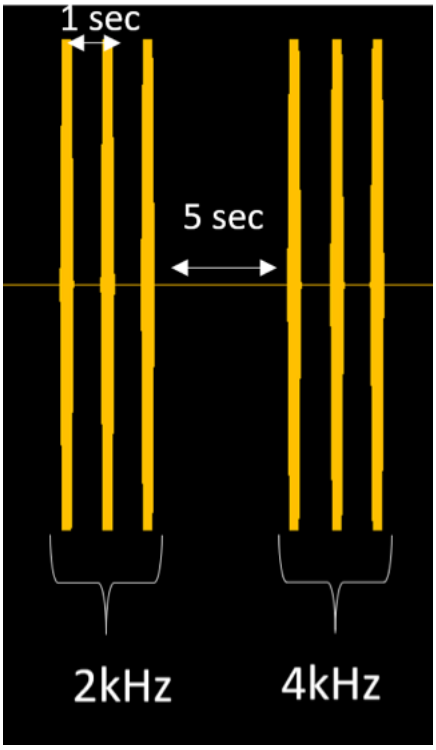

Figure S4: The sound stimulation.

The stimulation protocol was composed of 60db, three 500ms long sound pulses, spaced by 1000ms of silence. Each group of three pulses was of different frequency (between 0.2 to 15KHz, and separated from the previous group by 5000ms

Figure S5

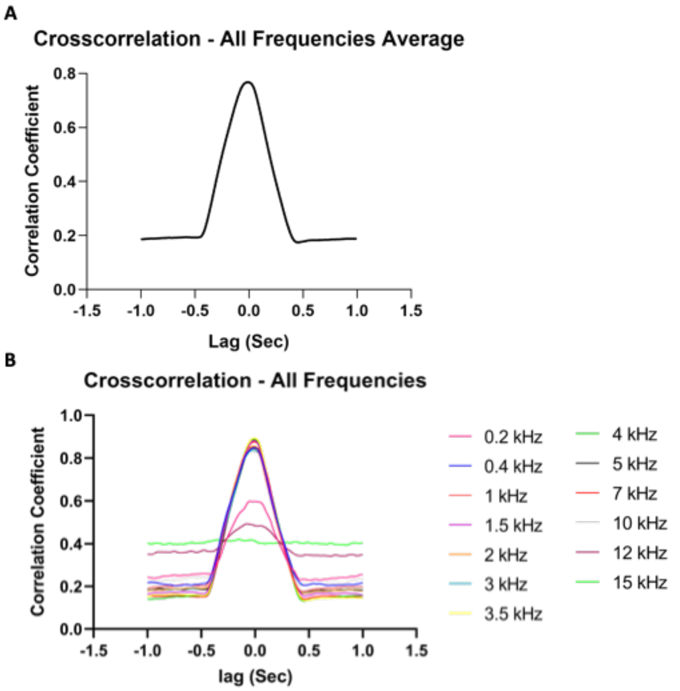

Figure S5: Cross correlation

Cross correlation between the stimulus and the electrophysiological response of the Ear-Chip. **(A)** present the average cross correlation of all the frequencies that were used to stimulate the Ear Chip. **(B)** present an overly of cross correlation for each frequency that was used to stimulate the Ear-Chip.

Figure S6

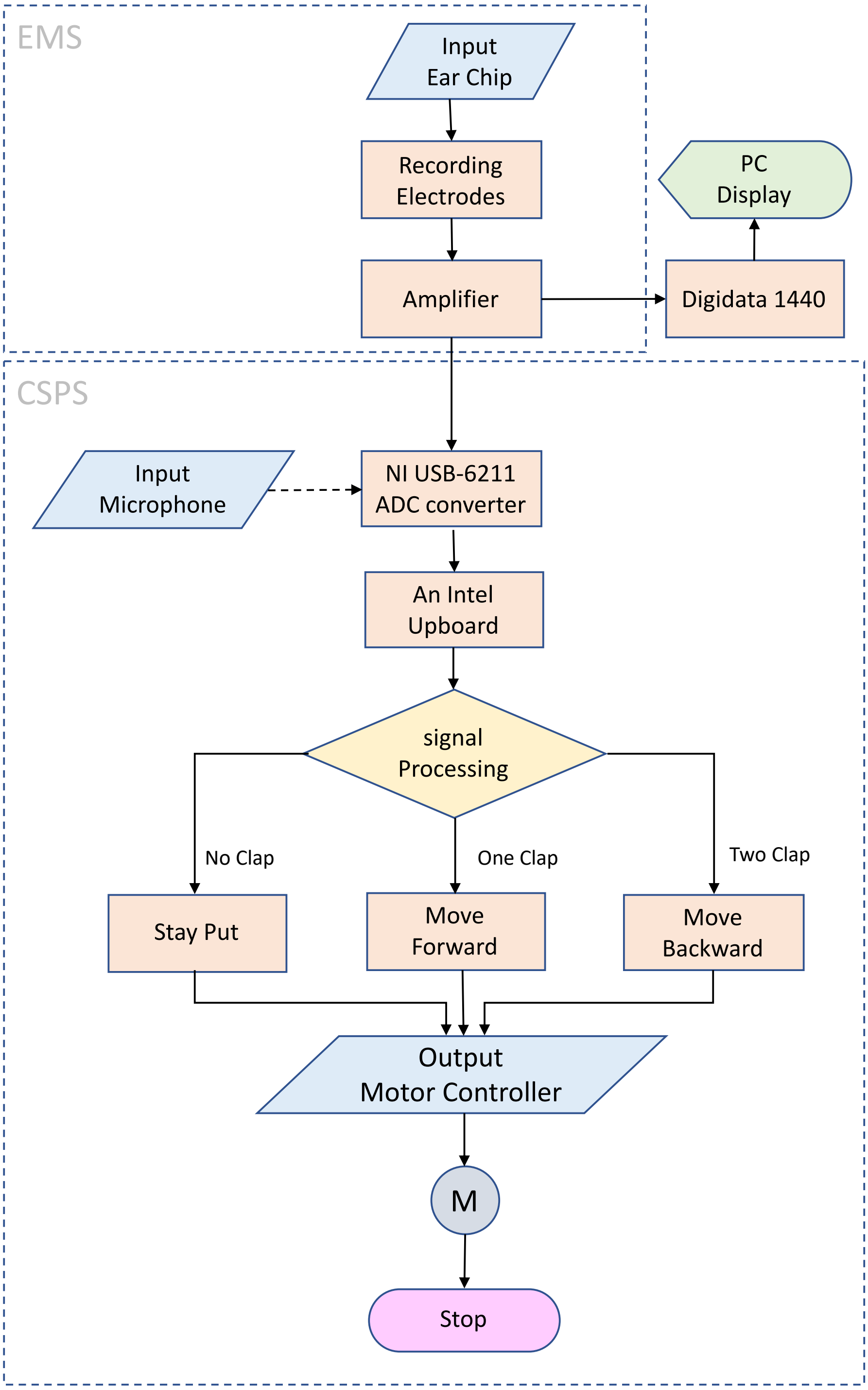

Figure S6

Logic diagram of the Ear-Bot.

Figure S7

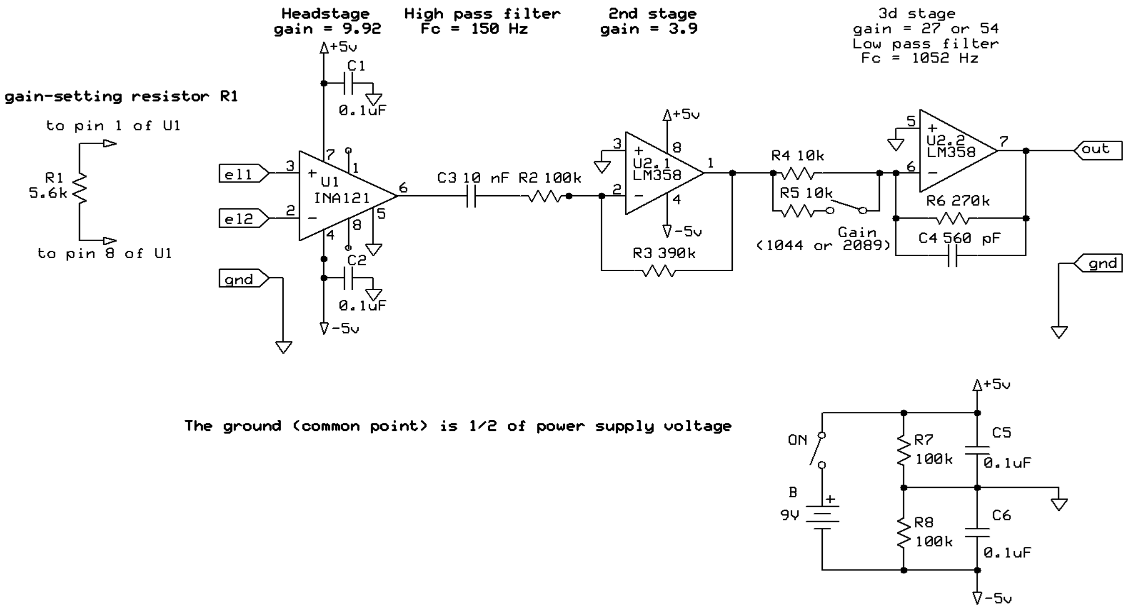

Figure S7: Schematics of the AC amplifier

AC amplifier schematics

SI movies:

Movie S1: Ear-Chip response to sound.

The locust tympanal organ integrated in the "Ear-Chip" and connected to the recording system. Top track on the computer screen shows the ear electrical response to a variety of sounds.

Movie S2: Custom platform for assessing the Ear-Chip spatial response to sound.

A custom-made system used to characterize the Ear-Chip response to sounds in different frequencies and different angles. The system custom holder enables to move the speakers both radially around the Ear-Chip and modify the distance from the Chip.

Movie S3: Ear-Bot response to sound.

The hybrid bio-robot moves forward as a response to one clap and backward as a response to two claps. The screen on the right shows the locust hearing organ's electrophysiological response to the claps.
